# Supplementary material for: Effects of nanoplastics on the gut microbiota of Pacific white shrimp Litopenaeus vannamei
Source: PeerJ. 2024 Jan 4;12:e16743. doi: 10.7717/peerj.16743 (PMC10771760; doi:10.7717/peerj.16743)
Supplement: Supplemental Information 1 [file peerj-12-16743-s001.docx]

Supplemental File

The following are the methods and raw data for Figure 4 for the determination of the relevant enzyme activities and biochemical indices.

**1. Biochemical parameters measure**

To measure antioxidant enzyme activity, livers were collected in each replicate and homogenized with 0.9 mL of 0.86% NaCl solution. The homogenate was centrifuged at 3500 rpm for 15 min at 4 °C. The supernatant was collected in sterile tubes for subsequent measurements. The protein concentration of the supernatant was determined using Komas Brilliant Blue. LZM, ACP, and AKP and concentration of TG, TCHO, and GLU content were measured with commercially available kits (Nanjing Jiancheng Bioengineering Institute, China). LZM（A050-1-1）, also known as muramidase or N-acetylmuramide glycanohydrlase, is an alkaline enzyme that hydrolyzes mucopolysaccharides in pathogenic bacteria. It lyses bacteria mainly by breaking the β-1,4 glycosidic bond between N-acetylmuramic acid and N-acetylamino glucose in the cell wall, causing the cell wall insoluble mucopolysaccharide to break down into soluble glycopeptides and leading to the escape of the cell wall rupture contents. ACP(A060-1-1), acid phosphatase breaks down disodium benzene phosphate and produces free phenol and phosphoric acid. Phenol interacts with 4-aminoantipyrine in alkaline solution and is oxidized by potassium ferricyanide to produce red quinone derivatives, and the enzyme activity can be determined according to the red color. AKP(A059-2-2), Alkaline phosphatase breaks down disodium benzene phosphate and produces free phenol and phosphoric acid. Phenol interacts with 4-aminoantipyrine in alkaline solution and is oxidized by potassium ferricyanide to produce a red quinone derivative, and the enzyme activity can be determined according to the shade of red. GLU(A154-1-1)，The plate was gently shaken and incubated at 37°C for 10 minutes at 505 nm, and the absorbance of each well was measured by an enzyme meter. TCH (A111-1-1) The color of the generated quinones was proportional to the cholesterol content, and the absorbance values of the calibration standard and sample tubes were measured separately to calculate the cholesterol content.

**2. Raw data for Figure 4**

| Table S1. The activity of LZM in CK, NP5, NP10, NP20 (U/mgprot). | | | |
| --- | --- | --- | --- |
| CK | NP5 | NP10 | NP20 |
| 136.2069 | 175.2069 | 195.4027 | 232.2309 |
| 141.9161 | 181.9161 | 186.6444 | 236.3438 |
| 153.4337 | 198.4337 | 194.0014 | 190.0074 |

Table S2. The activity of ACP in CK, NP5, NP10, NP20 (U/ml).

| CK | NP5 | NP10 | NP20 |
| --- | --- | --- | --- |
| 4.78 | 5.36 | 5.83 | 7.35 |
| 5.03 | 5.03 | 5.35 | 7.42 |
| 4.73 | 5.21 | 5.43 | 7.01 |

Table S3. The activity of AKP in CK, NP5, NP10, NP20 (U/ml).

| CK | NP5 | NP10 | NP20 |
| --- | --- | --- | --- |
| 4.78 | 4.36 | 4.83 | 6.35 |
| 4.03 | 5.03 | 4.35 | 7.42 |
| 4.73 | 5.21 | 5.43 | 7.01 |

Table S4. The content of TG in CK, NP5, NP10, NP20 (mmol/gprot).

| CK | NP5 | NP10 | NP20 |
| --- | --- | --- | --- |
| 0.3288 | 0.2705 | 0.2753 | 0.2512 |
| 0.3569 | 0.2326 | 0.3012 | 0.2305 |
| 0.3637 | 0.2216 | 0.2413 | 0.22075 |

Table S5. The content of TCHO in CK, NP5, NP10, NP20 (umol/gprot).

| CK | NP5 | NP10 | NP20 |
| --- | --- | --- | --- |
| 195.5688 | 181.6755 | 182.0954 | 142.3207 |
| 202.3325 | 172.9097 | 196.6915 | 169.7058 |
| 189.4243 | 168.9698 | 171.9125 | 154.175 |

Table S6. The content of GLU in CK, NP5, NP10, NP20 (mmol/gprot).

| CK | NP5 | NP10 | NP20 |
| --- | --- | --- | --- |
| 1.9041 | 1.8684 | 1.23 | 1.23 |
| 1.5474 | 1.6363 | 1.27083 | 1.07083 |
| 1.7756 | 1.3677 | 1.144 | 1.144 |

Table S7. Intestinal fold heights in four groups.

|  | Intestinal fold heights (μm) | Intestinal fold heights (μm) | Intestinal fold heights (μm) | Intestinal fold heights (μm) | Intestinal fold heights (μm) |
| --- | --- | --- | --- | --- | --- |
| CK | 160 | 161.5 | 156 | 155 | 170 |
| NP5 | 50 | 45 | 44 | 56 | 52 |
| NP10 | 40 | 41 | 46 | 44 | 39 |
| NP20 | 50 | 45 | 25 | 30 | 52 |

Figure S1. Statistical analysis of differences in intestinal fold height. *P* < 0.05 for NP5, NP10, and NP20 compared to the control group, ** indicates a significant *P* < 0.01 for NP5, NP10, and NP20 compared to the control group.
